# Critical role of hnRNP A1 in HTLV-1 replication in human transformed T lymphocytes

**DOI:** 10.1186/1742-4690-2-8

**Published:** 2005-02-09

**Authors:** Elsa Kress, Hicham Hachem Baydoun, Françoise Bex, Louis Gazzolo, Madeleine Duc Dodon

**Affiliations:** 1 Virologie Humaine INSERM-U412, Ecole Normale Supérieure de Lyon, IFR 128 Biosciences Lyon-Gerland, 46 allée d'ltalie 69364 Lyon Cedex 07, France; 2 Laboratory of Microbiology, University of Brussels, 1 Avenue E. Gryson, 1070 Brussels, Belgium

## Abstract

**Background:**

In this study, we have examined the role of heterogeneous nuclear ribonucleoprotein A1 (hnRNP A1) in viral gene expression in T lymphocytes transformed by HTLV-1.

**Results:**

We have previously observed that hnRNP A1 (A1) down-modulates the post transcriptional activity of Rex protein of HTLV-1. Here, we tested whether the ectopic expression of a dominant negative mutant (NLS-A1-HA) defective in shuttling activity or knockdown of the *hnRNPA1 *gene using RNA interference could inhibit Rex-mediated export of viral mRNAs in HTLV-1 producing C91PL T-cells. We show that the expression of NLS-A1-HA does not modify the export of Rex-dependent viral mRNAs. Conversely, inhibiting A1 expression in C91PL cells by RNA interference provoked an increase in the Rex-dependent export of unspliced and singly spliced mRNAs. Surprisingly, we also observed a significant increase in proviral transcription and an accumulation of unspliced mRNAs, suggesting that the splicing process was affected. Finally, A1 knockdown in C91PL cells increased viral production by these cells. Thus, hnRNP A1 is implicated in the modulation of the level of HTLV-1 gene expression in T cells transformed by this human retrovirus.

**Conclusions:**

These observations provide an insight into a new cellular control of HTLV-1 replication and suggest that hnRNP A1 is likely part of the regulatory mechanisms of the life cycle of this human retrovirus in T cells.

## Background

The human T cell leukemia/lymphotropic virus type 1 is the etiologic agent of adult T cell leukemia (ATL), an aggressive and fatal leukemia of CD4+ T lymphocytes [1,2] and is also associated with a neurological demyelinating disease, tropical spastic paraparesis (TSP) or HTLV-I associated myelopathy (HAM)[3]. Infection by HTLV-1 transforms T cells in vitro and in vivo, a process that has been associated with upregulation of specific cellular genes involved in T cell activation and proliferation during the course of viral infection [4-6]. The completion of the replication cycle of HTLV-1 leading to the production of new particles is dependent on two non-structural HTLV-1 encoded regulatory proteins, Tax and Rex, which act at the transcriptional and post-transcriptional levels, respectively [7,8]. The 40-kDa Tax protein trans-activates transcription of the provirus, through its interaction with cellular transcription factors and with Tax response elements present in the 5' long terminal repeat (LTR). The post-transcriptional activity of the 27-kDa Rex protein, an RNA-binding protein, is mediated by its interaction with the Rex response element (XRE) located on the U3/R region of the 3'LTR present on all viral transcripts [9]. When expressed at a critical threshold, Rex is able to direct the cytoplasmic expression of unspliced *gag-pol *and singly-spliced *env *mRNAs, at the expense of the multiply-spiced *tax/rex *mRNA [10,11]. We have recently reported that heterogeneous nuclear ribonucleoprotein A1 (hnRNP A1) interferes with the binding of Rex to the XRE, thus leading to a functional impairment of this viral protein [12].

The ubiquitously expressed hnRNP A1 is an abundant nuclear protein that participates in RNA processing, alternative splicing and chromosome maintenance as well as in the nucleocytoplasmic transport of mRNAs [13-18]. This protein contains two RNA-binding domains and a glycine-rich domain implicated in protein-protein interactions. Predominantly located in the nucleus, this cellular protein has the ability to shuttle continuously between the nucleus and the cytoplasm [19-21]. The signal that mediates both nuclear import and export has been identified as a 38-aa sequence, termed M9, located at the C-terminus of hnRNP A1, and is involved in the nucleo-cytoplasmic trafficking of mRNAs [22].

As indicated above, we have provided evidence that hnRNP A1 impairs the post-transcriptional regulation of HTLV-1 gene expression, by interfering with the binding of Rex to the XRE [12]. In the present study, we first demonstrate that the mutation of a putative binding site of hnRNP A1 to the XRE leads to an increase of the post-transcriptional activity of Rex. Next, to further address the effect that hnRNP A1 might exert on viral replication in vivo, we elected to investigate its implication in HTLV-1 producing T cells. Two experimental approaches were implemented: impairment of the functional activity of the endogenous hnRNP A1 by ectopic expression of a dominant negative mutant and knockdown of the hnRNPAl gene expression using RNA interference (siRNA). We report that inhibition of hnRNP A1 expression and functionality were achieved, leading to an increase of viral transcription together with an increase of cytoplasmic expression of viral mRNAs and of viral production. These observations by providing insight into a new cellular control of HTLV-I replication, suggest that hnRNP A1 is likely part of the regulatory mechanisms of the life cycle of this human retrovirus.

## Results

**Figure 1:**
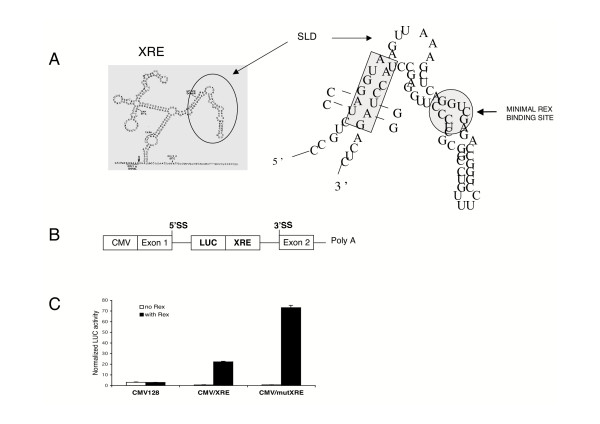
**Functional characterization of HTLV-1 mutated XRE sequence**. (A) Schematic representation of the HTLV-1 XRE. On the left, the XRE corresponds to U3 and R sequences within the HTLV-1 long terminal repeat, and consists of four stem-loops. On the right, the predicted secondary structure of the stem-loopD (SLD) with the minimal Rex binding site and the mutations introduced within the putative hnRNP A1 binding site are indicated. (B) Schematic view of the reporter plasmid CMV/XRE. (C) Effect of mutations within the XRE sequence on the Rex trans-activation capacity. Jurkat cells were transfected with 1 μg of the indicated reporter plasmid in the presence or not of Rex expression plasmid (200 ng) and the constitutive internal control tk-renilla luciferase vector (10 ng). Data are expressed as normalized luciferase activity and the error bars represent the standard deviations from three independent experiments.

A putative hnRNP A1 binding site has been identified, close to the minimal Rex binding site in the stem-loop D of the XRE (Fig 1A). To further evaluate the role of this binding site in the impairment of the functional activity of Rex, two punctual mutations were performed in the CMV/XRE vector containing the indicator *luc *gene (Fig 1B). These mutations modify the UAGGUA sequence into CCGGUA, and the UACCUA sequence into UACCGG, respectively, thus generating the CMV/mutXRE vector. Either vector (CMV/XRE and CMV/mutXRE), or the control vector (CMV 128, containing only the *luc *gene) were then transiently transfected in Jurkat cells in the absence or in the presence of a Rex-expressing plasmid. It was observed that, in presence of Rex, *luc *expression in cells transfected with the CMV/mutXRE vector was more than 3-fold higher than that in cells transfected with the CMV/XRE vector (Fig 1C). These results indicate that the putative hnRNPAl binding site close to the Rex binding site on the SLD sequence in the XRE is directly or indirectly implicated in down-modulating the post-transcriptional activity of Rex. Since the mutations affect a putative binding site for hnRNP A1, these results suggest that hnRNP A1 might be the effector of this down-regulation. To further delineate how this cellular protein perturbs the life cycle of HTLV-1, we elected to investigate its implication in HTLV-1 producing T cells. Two experimental approaches were implemented: impairment of the endogenous hnRNP A1 by ectopic expression of a dominant negative mutant (NLS-A1-HA) defective in shuttling activity and knockdown of the hnRNP A1 gene using RNA interference (RNAi).

### A nucleus-localized shuttling-deficient hnRNP A1 mutant does not affect the post-transcriptional activity of Rex

**Figure 2:**
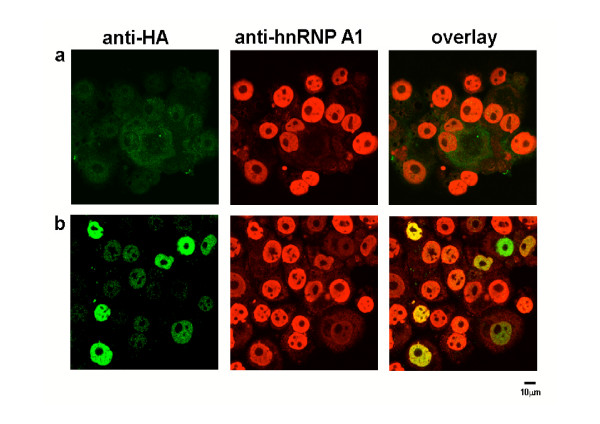
**Expression of a dominant negative mutant of hnRNP A1 in HTLV-1 producing C91PL cells**. Confocal microscopy of untransduced (a) or NLS-A1-HA transduced (b)-C91PL cells after dual immunofluorescence staining with anti-HA (green) and anti-hnRNP A1 (red) antibodies; the right panels show the overlay of the green and red staining;

The NLS-A1-HA construct contains the bipartite-basic type NLS of hnRNP K fused in frame with the N-terminus of an HA-tagged hnRNP A1 mutant, which lacked both nuclear import and export activities and inhibits hnRNP A1-dependent mRNA export when microinjected into nuclei of *Xenopus laevis *oocytes [22,23]. This hnRNP A1 mutant which retains the hnRNP A1 nuclear localization, lacks nuclear export activity [24]. As such, the nucleus-localized NLS-A1-HA has the potential to compete with wild-type hnRNP A1 for binding to mRNAs, and for its nuclear export. A retroviral vector LXSP-NLS-A1-HA was used to ectopically express this dominant negative mutant in the HTLV-1 transformed C91PL T cells. In these cells, Rex governs the cytoplasmic accumulation of unspliced (*gag/pol*) and singly-spliced (*env*) mRNAs. After a few days of culture in presence of puromycin, immunostaining of the resistant population revealed that about 30% of the cells were displaying HA labelling (Fig. 2). Dual immunostaining indicated that both endogenous hnRNP A1 (anti-hnRNP A1, red) and ectopically expressed NLS-A1-HA (anti-HA, green) displayed a nuclear diffuse staining excluding the nucleoli.

**Figure 3:**
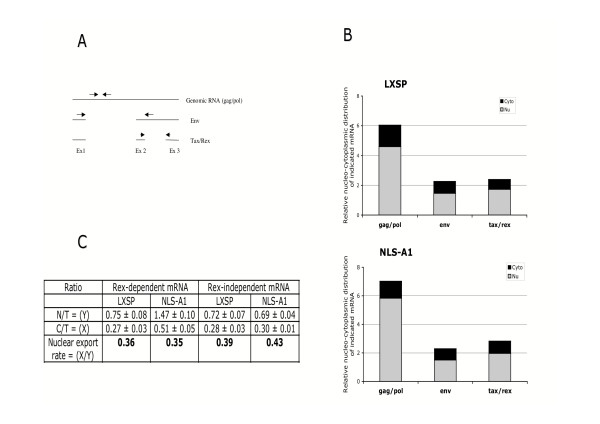
**Effect of ectopic expression of a dominant negative mutant of hnRNP A1 in HTLV-1 producing C91PL cells**. (A) Primer location on HTLV-1 mRNA; (B) Analysis of the nucleo-cytoplasmic distribution of viral gene expression in NLS-A1- and LXSP- transduced cells. Four days after transduction, mRNAs were extracted from the nuclear and cytoplasmic compartments of each cell type and levels of unspliced (*gag/pol*), singly spliced (*env*) and doubly spliced (*tax/rex*) mRNAs were reverse transcribed and quantified by real-time quantitative PCR (RQ-PCR), by using specific primers. Results are expressed as the amount of nuclear (grey bar) and cytoplasmic (black bar) indicated mRNA relative to β-actin. (C) Evaluation of the nuclear export rate (NER) of Rex-dependent (*gag/pol *plus *env*) mRNA and of Rex-independent (*tax/rex*) mRNA in NLS-A1- or LXSP- transduced C91PL cells. Numbers are the ratio between cytoplasmic (C) to total (T) RNA and nuclear (N) to total RNA.

We next investigated whether overexpression of this defective hnRNP A1 mutant was interfering with the expression of viral mRNAs. Quantification of the nuclear and the cytoplasmic levels of unspliced *gag/pol*, singly spliced *env *and doubly spliced *tax/rex *mRNAs was performed by RQ-PCR involving pair of primers specific of each viral mRNA (Fig. 3A). The comparative analysis of the viral mRNAs expression pattern between the control (LXSP) and NLS-A1-HA cells revealed a small increase of unspliced *gag/pol *and of doubly spliced *tax/rex *mRNAS in the latter, whereas no modification was observed for the singly spliced *env *mRNAs (Fig. 3B). The ratio of nuclear to total RNA and that of cytoplasmic to total RNA allowed to calculate a nuclear export rate (NER). Whereas the cytoplasmic expression of *tax/rex *mRNAs was slightly enhanced in cells expressing the NLS-A1-HA mutant, the NER of the unspliced and singly spliced mRNAs was not affected (Fig 3C). As the cytoplasmic expression of these mRNAs is Rex dependent, these results indicate that the ectopic expression of the NLS-A1-HA mutant in C91 PL cells does not interfere with the functionality of Rex. However and surprisingly, a more than 4-fold increase of the p19*gag *amount in the supernatant medium of NLS-A1-HA-transduced cells (2786 ± 154 pg/ml) was observed, when compared to the respective control cells (678 ± 104 pg/ml). Taken together, these results indicate that the impairment of the hnRNP A1 functionality might favour the translation of cytoplasmic viral mRNAs.

### Efficient inhibition of hnRNP A1 by retrovirus-delivered siRNAs

**Figure 4:**
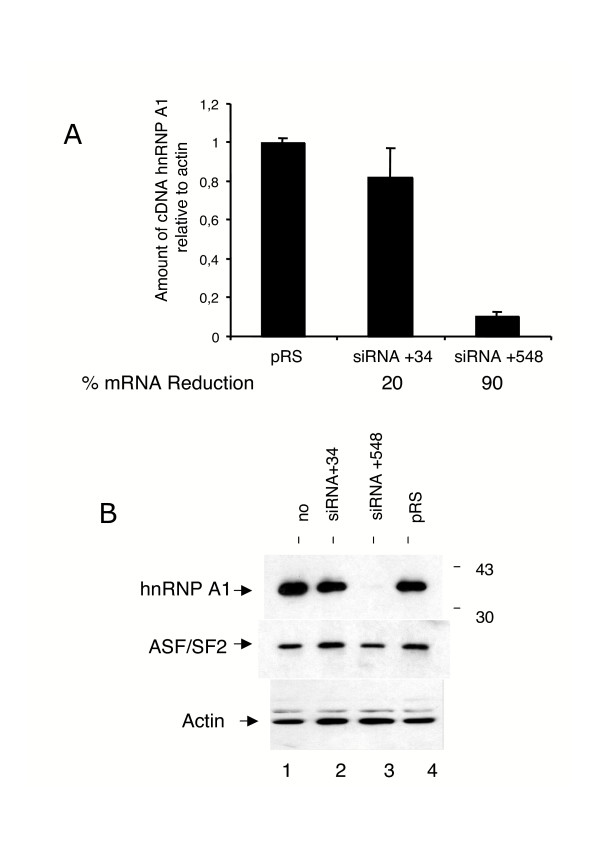
**RNAi-mediated reduction of hnRNP A1 expression in Jurkat cells**. (A) hnRNP A1 mRNA levels in cells transduced with the indicated retroviruses were determined by RQ-PCR. Levels in knockdown cells are given as percent mRNA reduction relative to the level in control cells transduced with empty pRS virus. Standard deviations are from at least three determinations performed in duplicate. (B) Equal amounts of protein from either nontransduced (lane1) or transduced with the indicated virus (lanes 2 to 4) were analyzed by immunoblotting. Actin and ASF/SF2 were used as control. Note that hnRNP A1 was significantly depleted in cells transduced with siRNA+548, whereas ASF/SF2 was not affected.

We next evaluated whether HTLV-1 replication is modulated by RNA interference with hnRNP A1 gene expression. To that aim, two oligonucleotides encoding siRNA directed against hnRNP A1, one targeting an RNA sequence located on the 5' end (34-nt after the translation start site), and the other an RNA sequence close to the 3'end (548-nt after translation start site) were each inserted in the pRS retroviral vector [25], as indicated in Materials and Methods. Both pRS-siRNA+34 and PRS-siRNA+548 vectors, as well as the pRS empty vector were used to produce recombinant retroviral particles used to transduce Jurkat T cells at a multiplicity of infection (m.o.i.) of 5. After four days of puromycin selection to eliminate nontransduced cells, the siRNA mediated-depletion of hnRNP A1 mRNAs was measured by quantitative RT-PCR. While targeting the 5'end (+34) was found inefficient, targeting the 3'end (+548) reduced the level of hnRNP A1 transcripts to 10% of those detected in untransduced Jurkat cells or in Jurkat cells transduced with empty (pRS) retroviral particles (Fig. 4A). Importantly, the siRNA-mediated reduction in A1 levels did not provoke cell death. Immunoblotting analysis of the PRS-siRNA +548 cells showed a strong reduction of the hnRNP A1 protein level, when compared to that in the pRS-siRNA+34 cells and in control cells (Fig 4B). Furthermore, the levels of the splicing factor ASF/SF2 were not modified in these cells. These data indicate that expression of hnRNP A1 is specifically repressed in the pRS-siRNA+548-transduced Jurkat cells.

### hnRNP A1 depletion in HTLV-1-producing T lymphocytes altered the transcriptional profile and increased the post-transcriptional activity of Rex

**Figure 5:**
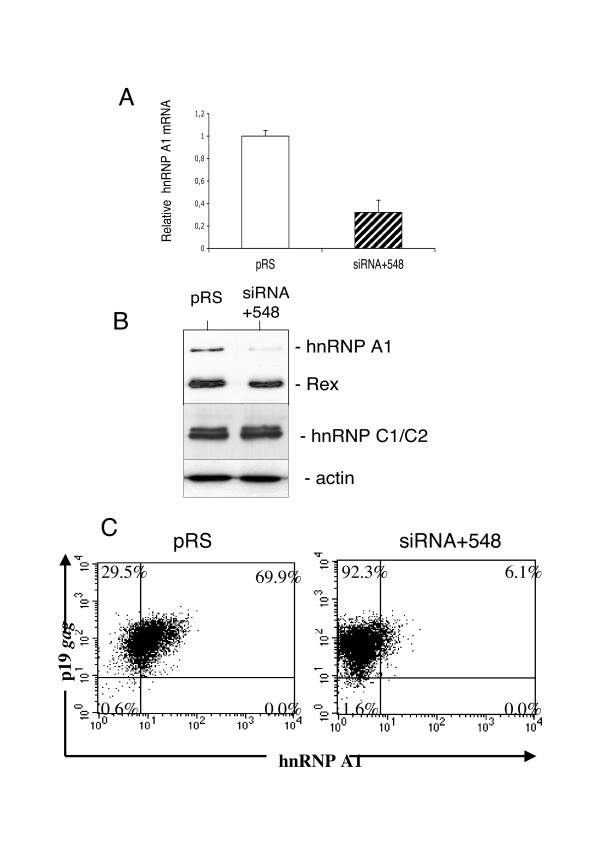
**Analysis of hnRNP A1 depletion in HTLV-1 producing C91PL cells**. (A) Analysis of hnRNP A1 mRNA levels in cells transduced with the indicated retroviruses. Four days after transduction, cytoplasmic RNA were extracted, reverse transcribed with oligo-dT, and levels of hnRNP A1 mRNA were determined by RQ-PCR. (B) Expression of hnRNP A1, Rex and hnRNP C1/C2 was monitored by immunoblotting of total protein extract from C91PL cells transduced with the indicated virus. Equivalent protein loading was confirmed by immunoblotting with an anti-actin antibody. (C) Detection of hnRNP A1 and p19*gag *expression in C91PL cells transduced with the indicated virus. Dot plots showing both hnRNP A1 and HTLV-1 gag expressions in one representative experiment. The percentage of cells in each quadrant is indicated.

The above described retroviral vector system was used to mediate the *in situ *synthesis of siRNAs and to suppress specifically *hnRNP A1 *gene expression in C91PL cells. Retroviruses produced from pRS-siRNA+548 and from the pRS empty vector were used to transduce these cells with a m.o.i. of 5. Four days after transduction, hnRNP A1 depletion was assessed by quantitative PCR analysis of cytoplasmic mRNAs. In siRNA-transduced C91PL cells, that transcript represented 32% of that in control pRS transduced cells (Fig. 5A). Interestingly, a western blot analysis of cell lysates further showed that hnRNP A1 was barely detected in siRNA-transduced C91 PL cells, whereas the levels of Rex, or of hnRNP C1/C2 or of actin were found unchanged (Fig. 5B). Furthermore, a flow cytometry analysis of siRNA-transduced C91PL cells reveals that hnRNP A1 was detected in 6.1% of these cells, whereas it was detected in about 70% of the control cells (Fig. 5C).

**Figure 6:**
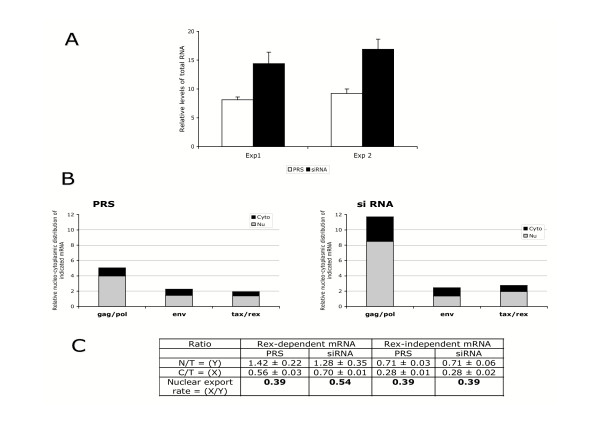
**Effect of hnRNP A1 depletion on viral gene expression**. (A) Quantification of total viral gene expression in siRNA-transduced C91PL cells by quantitative PCR. Nuclear and cytoplasmic mRNAs were extracted from siRNA (black bars)- or control PRS (white bars)- transduced C91PL cells. Equal amounts of mRNA were reverse transcribed with oligo-dT and subjected to RQ- PCR. Results are expressed as the relative levels of total viral mRNA to cellular β-actin. Error bars indicate standard deviations. (B) Analysis of the nucleo-cytoplasmic expression of viral genes. Four days after transduction, mRNAs were extracted and analyzed as in Fig. 3B. Results are expressed as the amount of nuclear (grey bar) and cytoplasmic (black bar) indicated mRNA relative to β-actin. (C) Evaluation of the nuclear export rate (NER) of Rex-dependent (*gag/pol *plus *env*) mRNA and of Rex-independent (*tax/rex*) mRNA in PRS- or siRNA- transduced C91 PL cells.

We next investigated whether the decrease in hnRNP A1 expression in C91PL cells was interfering with the expression of viral mRNAs. Real-time quantitative PCR assays were performed to quantify viral mRNAs by using the same primer pairs described above. Results (from two different transduction experiments) assessing the amount of total viral mRNAs (Fig 6A) revealed that suppression of hnRNP A1 in siRNA-transduced C91PL cells was leading to a significant increase of viral transcription (1.7 to 1.8 fold), when compared to PRS control cells. Then, the analysis of the relative nuclear and cytoplasmic levels of unspliced *gag/pol*, singly spliced *env *and doubly spliced *tax/rex *mRNAs indicated that the expression of unspliced gag/pol mRNA was 2 and 3-fold enhanced respectively in the nucleus and cytoplasm of siRNA-transduced C91PL cells, whereas the expression and the distribution of spliced env mRNAs were not significantly altered (Fig. 6B). A slight increase of the doubly-spliced *tax/rex *mRNAs was observed in both compartments.

These results suggest that inhibition of hnRNP A1 in C91PL cells mainly correlates with a defect in the splicing of genomic mRNAs. The NER of the unspliced and singly spliced mRNAs was significantly higher in siRNA-treated cells than in control cells, whereas the cytoplasmic expression of tax/rex mRNAs, which is Rex-independent was not modified (Fig. 6C). As the nucleo-cytoplasmic transport of the former is Rex-dependent, these observations propose that the depletion of hnRNPAl correlates with an increase of Rex activity. Finally, whereas a flow cytometry analysis indicated a similar percentage of p19*gag *producing cells in siRNA-transduced C91PL cells and in control cells, the quantification of 19*gag *in the supernatant medium of siRNA-transduced cells revealed a 1.5-fold increase of the p19*gag *amount (1017 ± 26 pg/ml), compared to that in control cells (678 ± 104 pg/ml).

Collectively, these data support that the hnRNP A1 depletion in HTLV-1-producing T cells increases viral transcription, is correlated with a defect in the splicing process at the level of the *gag/pol *transcript and increases the post-transcriptional activity of Rex leading to an increase of viral production.

## Discussion

The ubiquitously expressed hnRNP A1, an RNA-binding protein, is a nucleocytoplasmic shuttling hnRNP that accompanies eukaryotic mRNAs from the active site of transcription to that of translation. As such, hnRNP A1 is involved in a variety of important cellular functions, including RNA splicing, transport, turnover and translation. We have previously shown that hnRNP A1 decreases the post-transcriptional activity of the Rex protein of HTLV-1, by interfering with the binding of the viral protein on its response element, present on the 3' LTR of all viral RNAs. Here we first report that the mutation of a putative binding site of hnRNP A1 in the XRE enhances the functional activity of Rex. This observation obtained through transient transfection experiments, confirms that A1 proteins could antagonize the post-transcriptional activity of Rex, by a competitive mechanism.

We have next investigated the role of hnRNP A1 in HTLV-1 transformed C91PL cells, which produce HTLV-1 virions. These express the three differentially spliced (the unspliced *gag/pol*, the singly spliced *env *and the doubly spliced *tax/rex*) mRNAs, which encode the structural and regulatory proteins. The *gag/pol *and *env *mRNAs are dependent on Rex for their cytoplasmic expression. To determine whether hnRNP A1 interferes with viral replication, we first examined the effect of the ectopic expression of an hnRNP A1 mutant (NLS-A1-HA) defective in nuclear export activity. This mutant was previously used to assess the potential role of hnRNP A1 in nucleocytoplasmic shuttling activity in normal and leukemic myelopoiesis. Interestingly it was found that the ectopic expression of this dominant negative form of hnRNP A1 resulted in the downmodulation of the nucleocytoplasmic trafficking of cellular mRNAs that encode proteins affecting the phenotype of normal and transformed myeloid progenitors [24]. In the present study, we showed that NLS-A1-HA- C91PL cells expressed a higher level of total viral transcripts than that observed in control cells, suggesting that the ectopic expression of this hnRNP A1 mutant correlated with an increased proviral transcription and/or stability of the viral RNA.

Furthermore, no modification of the nuclear export rate was observed in the NLS-A1-HA-transduced C91PL cells, indicating that the activity of Rex was not impaired. Finally, as both endogenous hnRNP A1 and the NLS-A1-HA mutant, which are nucleus-localized and consequently able to access the XRE did not decrease the Rex-dependent nucleo-cytoplasmic expression of the viral mRNAs, we should therefore speculate that the simultaneous presence of both types of A1 forbids them to bind the XRE with maximal efficiency. Interestingly, the increase of p19*gag *produced by the NLS-A1-HA C91PL cells suggests that the retention of the endogenous hnRNP A1 in the nucleus is favouring an increase in the translation of viral mRNAs

We have then proceeded to the knockdown of hnRNP A1 gene using the retrovirus-mediated RNA interference. This system was first validated in transduction experiments performed in Jurkat T cells. A puromycin-selected population of cells was obtained in which a strong overall specific reduction of hnRNP A1 was observed. Note that this hnRNP A1-depleted Jurkat cells were not affected in their growth even for a long time culture (data not shown). This is consistent with other studies showing that si-RNA-mediated reduction in A1 levels did not affect cell division nor provoke cell death in normal cell lines [26].

We next performed siRNA depletion of hnRNP A1 in C91PL cells and have observed a significant increase in proviral transcription, as demonstrated by the higher level of viral transcripts than that in control cells (Figure 6A). Furthermore, the level of unspliced transcripts was found to be predominant, compared to the singly-and doubly-spliced transcripts, in the hnRNP A1 depleted cells, pleading for a splicing default (Fig. 6B). Finally, the increase of the nuclear export of unspliced and singly spliced mRNAs suggests that the knockdown of hnRNP A1 allows a better accessibility of Rex to the XRE and leads to the enhancement of the post- transcriptional activity of Rex. This is in good correlation with the increase in the production of viral particles, as ascertained by the quantification of the p19*gag *protein. Since hnRNP A1 has been implicated in nuclear export of cellular mature mRNAs [27] as well as translational and/or posttranslational events of viral mRNAs (our study), it is possible that its depletion could affect the expression of several transcription and/or splicing factors, leading to an effect, for instance, on the splicing process of viral mRNAs.

Of the two experimental approaches used in the present study to apprehend the implication of hnRNP A1 on HTLV-1 replication in *in vitro *HTLV-1-transformed T-cells, that consisting in the depletion of this cellular protein by RNA interference provides evidence for the role of hnRNP A1 in restraining the viral life cycle at both transcriptional and post-transcriptional levels. We conclude from these findings that down-regulation of hnRNP A1 has an important role on the replicative potential of HTLV-1 in T lymphocytes. Consequently, these data allows us to define hnRNP A1 as a cellular protein endowed with an anti-HTLV-1 activity.

## Methods

### pRS construct directing the synthesis of siRNA and Plasmids

The vector pRetro-SUPER (pRS) was used to generate biologically active siRNAs from the Pol III H1-RNA gene promoter [25]. Two annealed 64-bp synthetic oligonucleotides were used: 5'-gatccccAGCAAGAGATGGCTAGTGCttcaagagaGCACTAGCCATCTCTTGCTtttttgga aa-3', and 5'-gatccccCAGCTGAGGAAGCTCTTCAttcaagagaTGAAGAGCTTCCTCAGCTGtttttgga aa-3'. The sequence of each oligonucleotide was designed (Oligoengine) to encode two 19-nt (in capital letters) reverse complements homologous to a portion of hnRNP A1 (nucleotides 34–53 for the first construct, and nucleotides 548–567 for the second one) separated by a 9-nt spacer region, and ending by Bgl II and Hind III sites. Each oligonucleotide was then introduced into pRS resulting in either pRS-siRNA+34 or pRS-siRNA+548 retroviral vectors, respectively. Plasmids pgagpol/MLV and EnvVSV-G were kindly provided by F.L. Cosset (U412-Lyon). LXSP-NLS-A1-HA and empty LXSP retroviral vectors were a kind gift of D. Perrotti and has been described previously [23,24].

For reporter gene analyses, the luciferase plasmid (CMV/XRE) was derived from the reporter plasmid pDM138 containing the CAT gene and the XRE sequences [28]. It expresses, under the control of the cytomegalovirus promoter, a two-exon, one-intron precursor RNA in which the *luc *gene and the *XRE *are located within the intron (see Fig. 1B). The mutant plasmid (CMV/mutXRE) was generated using a site-directed mutagenesis kit (Stratagene) according to the manufacturer's instructions, and with the following primer, 5'-AAAGCCCTGTCAAAACAGGAAATGGCAAGCGCTTCATCCAGCC-3'. This construct was verified by DNA sequencing before use in transfection. The *rex-*expression plasmid, containing the wild type Rex sequence under the control of the cytomegalovirus promoter, was a gift from B.C. Cullen.

### Cell culture and DNA transfection

Jurkat lymphoblastoid T-cells were incubated at 37°C in a 5% CO2 atmosphere, in RPMI-1640 medium (Invitrogen) supplemented with 10% heat-inactivated fetal calf serum (FCS) and 20 IU/ml penicillin, 20 μg/ml streptomycin. The HTLV-1-transformed T-cell line, C91PL [29] was cultured in complete RPMI medium. The human epithelial 293T cells and the human rhabdomyosarcoma TE cellswere cultured in Dulbecco's minimum eagle medium (DMEM, Invitrogen) supplemented with 10% FCS and 20 IU/ml penicillin, 20 μg/ml streptomycin. These cells seeded at 1.2 × 10^5 ^cells per well of a 12-well plate were transfected using the calcium phosphate coprecipitation technique [30]. Jurkat cells were transfected by using the X-treme GENE Q2 transfection reagent (Roche Molecular Biochemicals) according to the manufacturer's indications. The amount of plasmid used in each transfection assay is indicated in the figure legends. To assess the efficiency of the transfection assay, 10 ng of the tk-renilla Luciferase plasmid (Promega) were co-transfected in each assay. Cells were harvested 24 h after transfection, resuspended in 100 μl of passive lysis buffer (Promega) and assayed for both firefly and renilla luciferases by using a Dual-Luciferase Reporter assay system (Promega).

### Preparation of viral stocks and transduction of T cells

Fresh viral stocks were prepared by transfecting 293T cells (seeded at 5 × 10^5 ^cell/well of a 6-well plate) with 2 μg of pRS or pRS-siRNA together with 1 μg of pgag-pol/MLV and 0,45 μg of env/VSV-G with ExGen 500 reagent (Euromedex). Twelve hours later, the cells were washed once with PBS, and newly produced virions were harvested over 24 h in 1,5 ml of fresh medium. Viral supernatants were clarifed by passage through a 0.45-μm syringe filter and aliquots were stored at -80°C. Titers of virus stocks were determined by infecting rhabdomyosarcoma human TE cells (60% confluent) with serially diluted viral stocks. After infection, cells were split and plated in the presence of puromycin (5 μg/ml); puromycin-resistant colonies were scored after 7 days. Virus titers generally ranged from 3 to 5 × 10^5 ^transducing units per ml.

Transduction of Jurkat or of C91 PL T cells with retroviral vectors was carried out as followed: briefly, cells (1 × 10^6^) plated in a 24-well plate were infected at a multiplicity of infection (moi) of 5 with viral stocks in a final volume of 1.0 ml containing 4 μg of polybrene/ml, for 18 h and allowed to recover for 24 hr with fresh medium. When necessary, transduced cells were selected with puromycin 4–5 μg/ml for 4 days and maintained in culture for long time period with 1 μg/ml puromycin.

### RNA isolation and real time quantitative RT-PCR

Nuclear and cytoplasmic RNAs were extracted from 2 × 10^6 ^cells by using an Rneasy RNA-preparation kit (Qiagen) according to the manufacturer's instructions. To reduce the amount of DNA originating from lysis, samples were treated with Rnase-free Dnase (10 U/μl, Boehringer) for 30 min at 20°C and then for 15 min at 65°C. 500 ng of RNA sample were reverse transcribed by using oligo(dT)12–18 and Superscript II (Life Technologies, Inc.). Reverse transcription was performed for 50 min at 42°C. The total cDNA volume of 20 μl was frozen until real-time quantitative PCR was performed. After thawing for PCR experiments, the cDNA was diluted in distilled water and 2 μl of diluted cDNA was used for each PCR reaction. The realtime quantitative PCR (RQ-PCR) was performed in special lightcycler capillaries (Roche) with a lightcycler Instrument (Roche), by using the LightCycler-FastStart reaction Mix SYBR-Green kit (Roche). The following specific primers were used to detect: hnRNP A1, sense 5'-AAGCAATTTTGGAGGTGGTG-3' and antisens, 5'-ATAGCCACCTTGGTTTCGTG-3', gag/pol_HTLV-1_sense, 5'-CCCTCCAGTTACGATTTCCA-3' and antisens, 5'-GGCTTGGGTTTGGATGAGTA-3', env_HTLV-1_sense, 5'-CTGTGGTGCCTCCTGAACT-3' and antisens, 5'-AAAGTGGCGAGAAACTTACCC-3', pXIII sense, 5'-ATCCCGTGGAGACTCCTCAA-3' and antisens, 5'-CCAAACACGTAGACTGGGTATCC-3'. β-actin sense,5'-TGAGCTGCGTGTGGCTCC-3' and antisens: 5'-GGCATGGGGGAGGGCATACC-3'.

The thermal cycling conditions consisted of 40 cycles at 95°C for 10 sec, 61°C for 5 sec, 72°C for 10 sec. The fluorescence signal increase of SYBR-GREEN was automatically detected during the 72°C phase of the PCR. Omission of reverse transcriptase in the RT-PCR protocol led to a failure of target gene amplification in the positive controls. Light cycler PCR data were analyzed using LightCycler Data software (Idaho Technology). The software first normalizes each sample by background subtraction of initial cycles. A fluorescence threshold is then set at 5% full scale, and the software determines the cycle number at which each sample reached this threshold. The fluorescence threshold cycle number correlates inversely with the log of initial template concentration. β-actin transcript levels were used to normalize the amount of cDNA in each sample. Melting curve profiles were used to confirm amplification of specific transcripts.

### Immunoblotting

Cells were washed and harvested in ice-cold PBS containing protease inhibitors (complete mini EDTA-free, Roche Molecular Biochemicals). Cells were lysed in RIPA buffer (150 mM NaCI, 50 mM Tris-HCI pH 8.0, 0.5% deoxycholate, 0.1% SDS, 0.5% Nonidet P-40, protease inhibitors, 80 U/ml endonuclease) and incubated for 30 min at 4°C. After centrifugation at 12,000 rpm for 10 min at 4°C, the supernatant was assayed for protein content by Bradford assay (Bio-Rad). Equal amounts of proteins were separated by SDS/PAGE.

Cells were lysed in Laemmli buffer and equal amounts of proteins were subjected to 12% SDS-PAGE. They were subsequently blotted onto nitrocellulose membrane (BA, Schleicher & Schuell). The membrane was then blocked overnight at 4°C in blocking buffer (PBS and 0.1% Tween-20) supplemented 10% non-fat powdered milk and probed with the appropriate antibody diluted in blocking buffer plus 10% non-fat powdered milk. The following antibodies were used: rabbit anti-actin (Sigma), mouse anti-ASF/SF2 (gift from Dr. J. Stevenin) mouse monoclonal anti-hnRNP A1 and anti-hnRNP C antibodies (4B10 and 4F4, respectively; gifts from G. Dreyfuss), followed with an anti-rabbit (Immunotech, France) or anti-mouse (Dako) Immunoglobulin G-horse radish peroxidase-conjugated antibody. Blots were then developed using an enhanced chemiluminescence detection system (Renaissance, NEN, Life Science Products). Bands were visualized by using Hyperfilm (Amersham Pharmacia Biotech).

### Flow cytometric analysis and Immunostaining

Cells (5 × 10^5^) were washed twice with PBS, resuspended in 3% (vol/vol) paraformaldehyde/PBS for 45 min at room temperature, and permeabilized with 0.5% Triton X-100/PBS for 5 min. After washing with PBS, the cells were incubated with specific antibodies (4B10) diluted in 1% BSA/PBS for 1 h. Cells were washed twice with PBS and were then incubated with FITC-conjugated goat anti-mouse, PE-conjugated goat anti-rabbit in 1% BSA/PBS for 40 min. Cells were washed three times with PBS and resuspended in a 2% paraformaldehyde/PBS solution. The fluorescence intensity was measured on a FACScan instrument (Becton Dickinson Labware, Mountain View, Calif;). The integrated fluorescence of the gated population was measured, and data from 10,000 analyzed events were collected.

For immunostaining, C91PL cells were centrifuged on cytoslides using a cytospin (Thermo Shandon, Pittsburgh, PA), fixed on slides with 3.7% paraformaldehyde for 15 min at room temperature, and permeabilized with 0.5% Triton X100 for 5 min in 4°C. The samples were saturated with PBS containing 0.5% gelatin and 0.25% bovine serum albumin for 1 h and stained for 1 h with a 1/100 dilution of a rabbit polyclonal serum directed against HA (Y11 from Santa Cruz Biotechnology) (NLS-A1-HA staining) or 1/1000 dilution of mouse monoclonal antibodies (4B10) (hnRNP A1 staining) in the same saturation solution. The samples were then washed three times with PBS containing 0.25% gelatin and incubated for 1 h with a 1/100 dilution of the following secondary antibodies: goat anti-rabbit immunoglobulin G conjugated to fluorescein isothiocyanate (green color for HA) and goat anti-mouse immunoglobulin G conjugated to lissamine rhodamine sulfchloride (red color for hnRNP A1) (Jackson Immunoresearch). The samples were washed three times in PBS with 0.25% gelatin and mounted for analysis on a Zeiss LSM 510 laser scanning confocal microscope.

### ELISA

p19gag was measured in culture medium using the RETROTEK HTLV p19 Antigen ELISA kit (Zeptometrix). Medium of the cell culture was centrifuged at low speed to remove the cell debris, and filtrated through a 0,45-μm filter. The amount of Gag protein was quantified in the resultant supernatant according to the manufacturer procedure. Results are expressed as pg/ml of p19 protein and are the mean of two different experiments, each point tested in quadruplicate.

## Competing interests

The author(s) declare that they have no competing interests.
